# Comparing the Effectiveness of Glucocorticoids in Preventing Hypertrophic Scar Diagnosis in Burn Patients

**DOI:** 10.3390/medicina59111970

**Published:** 2023-11-08

**Authors:** Alejandro Joglar, Juquan Song, George Golovko, Jayson Jay, Steven Wolf, Amina El Ayadi

**Affiliations:** Department of Surgery, University of Texas Medical Branch, Galveston, TX 77555, USA; aajoglar@utmb.edu (A.J.); jusong@utmb.edu (J.S.); gegolovk@utmb.edu (G.G.); jwjay@utmb.edu (J.J.); swolf@utmb.edu (S.W.)

**Keywords:** glucocorticoid, hypertrophy, hypertrophic scar, burn, burn patients, TriNetX

## Abstract

*Background and Objectives*: The prevalence of hypertrophic scarring after a burn is approximately 70%. Despite advances in burn management, there is currently no gold standard treatment to reduce or prevent its occurrence. Glucocorticoids are frequently given to patients early after burns for other therapeutic purposes and have been shown to induce scar regression. Therefore, the purpose of the present work is to determine the incidence of hypertrophic scar diagnosis in burn patients who were administered glucocorticoid treatment using TriNetX, a large patient database. *Materials and Methods*: Patients diagnosed with hypertrophic scarring, hypertrophic disorders of the skin, or scar conditions and fibrosis of the skin after burn injury were identified in the TriNetX database. The glucocorticoids investigated include hydrocortisone, methylprednisolone, dexamethasone, triamcinolone, and prednisone. Patients were stratified into three groups based on total body surface area (TBSA) burned: 0–19%, 20–39%, and 40–100%. The risk ratio was evaluated for burn patients who received varying glucocorticoids after injury based on TBSA burned. Additionally, treatment pathways, time of treatment, and treatment purity pathways were evaluated. *Results*: In patients with a 0–19% TBSA burn, methylprednisolone showed a decreased risk of developing hypertrophic scar diagnosis. In those with a 20–39% TBSA burn or 40–100% TBSA burn, dexamethasone showed an increased risk of developing hypertrophic scar diagnosis. Additionally, dexamethasone was the most commonly administered glucocorticoid for burn patients and was most likely to be administered earlier after burn injury, comparatively. *Conclusions*: Methylprednisolone was associated with reduced hypertrophic scar diagnosis in burn patients independent of TBSA burn. While glucocorticoids are one of the mainstay treatments for hypertrophic scarring, further studies are needed to determine early therapeutic interventions that will reduce the potential for hypertrophic scar development in burn patients.

## 1. Introduction

Hypertrophic scars are defined as elevated scars that remain within the boundaries of a wound, may develop within 4 to 8 weeks, and may regress spontaneously. The prevalence of hypertrophic scarring after a burn injury is approximately 70%. and may cause physical and mental injury to patients by causing physical limitations and affecting their appearance [1].

Despite advances in burn management where patients can survive up to 100% total body surface area (TBSA) burned, no optimal treatments to reduce or prevent hypertrophic scars are currently available [2]. Current treatments for hypertrophic scars include surgery, cryotherapy, pressure therapy, glucocorticoid injections, and laser therapy. These treatments are painful, costly, have a high scar recurrence rate, and may fail to reduce scarring after the initial intervention [2]. Additionally, these treatments are focused on treating the scars rather than preventing scar formation. Thus, there has been an increase in research studying potential pharmacologic treatments for preventing the development of hypertrophic scars. 

Glucocorticoids are frequently given to burn patients early after a burn injury for several therapeutic purposes. Burn patients experience a significant inflammatory response after a burn injury, and glucocorticoids help mitigate this early inflammatory response. Burn patients who received glucocorticoids showed reduced risk of pulmonary infections, acute respiratory distress syndrome, stress ulcers, and length of stay in hospital [3]. Additionally, glucocorticoids decreased the risk of developing hypertrophic scars in burn patients [3].

The early inflammatory response is characterized by a surge in pro-inflammatory cytokines. Among those, TGF-beta is known to induce the differentiation of dermal fibroblasts into muscle-like fibroblasts, or myofibroblasts. This process is essential for wound closure but can go uncontrolled with the extended inflammatory response seen in burn patients [4]. 

During the normal wound healing process, myofibroblasts are recruited to produce extracellular matrix (ECM) and assist with wound contraction. Hypertrophic scars occur due to an increase in the production of ECM (ex. Type 1 and 3 collagen) by the myofibroblasts, which are found in excess in hypertrophic scars [5]. Due to poor regulation of type 1 collagen, there is a higher ratio of type 1 to type 3 collagen in hypertrophic scars [6]. It has been shown that after a burn injury, the inflammatory response occurs immediately and may persist for several months [6]. This pathophysiologic response leads to poor wound healing and increased fibroblast activity, leading to an increase in ECM deposition [6]. Glucocorticoids may reduce hypertrophic scars through their ability to reduce collagen production, inflammatory mediators, and fibroblast proliferation [7]. 

The purpose of the present work is to determine the incidence of hypertrophic scar diagnosis in burn patients who were administered glucocorticoid treatment using a large patient database. We investigated the main glucocorticoids used in burn settings, including methylprednisolone, hydrocortisone, dexamethasone, and triamcinolone. Treatment pathways and length of treatments were also identified among the various glucocorticoids studied. 

## 2. Materials and Methods

Burn patients (ICD–10 code T30-T31) were identified using the TriNetX database. TriNetX is a North American federated health research network that provides de-identified electronic health record information for over 40 million patients in 55 partner healthcare organizations, including hospitals, primary care, and specialty providers. Relevant patient information provided by TriNetX includes diagnosis, health outcomes, medications, procedures, and demographics. IRB approval was exempt for this retrospective study because Protective Health Information or Personal Data was not used and TriNetX is HIPPA and GDPR compliant (IRB Exemption #20-0085).

**Figure 1:**
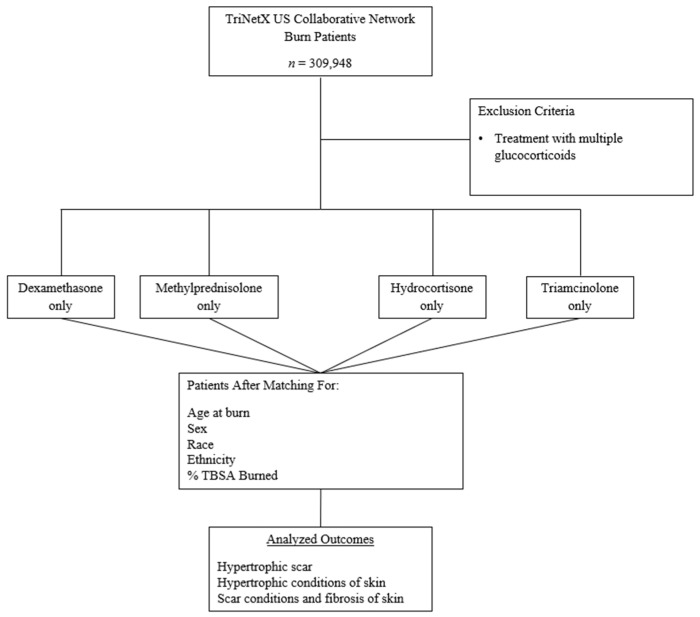
A stepwise representation of the study design shows the inclusion-exclusion criteria, treatments, group selection, and outcome analysis.

Patients who were diagnosed with hypertrophic scarring (ICD–10 code L91.0), hypertrophic disorders of the skin (ICD–10 code L91), or scar conditions and fibrosis of the skin (ICD–10 code L90.5) after burn injury were identified. Hydrocortisone (ICD–10 code 5492), methylprednisolone (ICD–10 code 6902), dexamethasone (ICD–10 code 3264), and triamcinolone (ICD–10 code 10759) were the four glucocorticoids investigated. Patients who were administered glucocorticoids on the same day or at any time within 10 months of a burn injury were compared to those who did not take any of the five glucocorticoids within five years of a burn injury. Patients were then stratified into three groups based on percent total body surface area (TBSA) burned: 0–19%, 20–39%, and 40–100%. The control group consisted of a total of 165,041 burn patients who did not receive any of the five glucocorticoids after experiencing a burn injury. The method of administration for each glucocorticoid was not specified when comparing each group. Figure 1 represents the step-wise method of how each cohort was constructed in TriNetX. 

Each patient cohort was balanced based on age of index, ethnicity, race, and sex. The age of index was defined as the age at which patients were diagnosed with the investigated diagnosis. Ethnicity was defined as whether the patient was classified as Hispanic or Latino, not Hispanic or Latino, or an unknown ethnicity. Race was defined as whether a patient was classified as White, Black, or African American; unknown race; American Indian or Alaska Native; Native Hawaiian or Other Pacific Islander; or Asian. Sex was defined as patients who identified as male, female, or unknown. Table 1 represents the demographics of the patient population identified in the study. 

Statistical analysis: Group comparison was conducted using the TriNetX analytical tools package to determine risk ratios, odds ratios, incidence, and treatment pathways. Risk ratios with a *p*-value < 0.05 were considered significant. 

## 3. Results

### 3.1. Effect of Glucocorticoids on Burn Outcomes

Hydrocortisone administration between 0 and 10 months after burn increased the risk of developing hypertrophic scar diagnosis in burn patients with a 0–19% TBSA burn (Table 2). An increased risk of scarring was also observed in patients administered triamcinolone or dexamethasone. However, patients given methylprednisolone showed a decreased risk of developing hypertrophic scar diagnosis. 

No significant effects on scarring were observed in burn patients with a TBSA of 20–39% who received hydrocortisone, triamcinolone, or methylprednisolone. However, patients in this TBSA group given dexamethasone showed an increased risk of developing hypertrophic scars. 

Similarly, no significant risk of developing hypertrophic scarring was observed in burn patients with a TBSA of 40–100% when given hydrocortisone, triamcinolone, or methylprednisolone. However, this risk increased significantly in burn patients given dexamethasone. (Table 2).

### 3.2. Glucocorticoid Treatment Pathways in Overall Burn Patients

**Figure 2:**
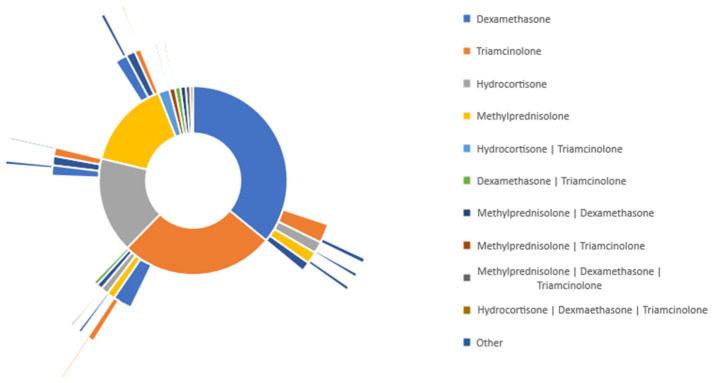
Glucocorticoid treatment pathway in all burn patients (TBSA unspecified).

To determine the glucocorticoid treatment distribution in burn patients, we used the treatment pathway analytical tool in TriNetX, which allows for the identification of patients’ medications over time. Figure 2 represents the treatment pathway of all burn patients who received either of the four specified glucocorticoids. Dexamethasone was the most commonly administered glucocorticoid in burn patients, with 36.1% of patients receiving this specific medication. Triamcinolone was the second most common glucocorticoid, with 25.7% of patients receiving the medication. 15.90% of patients received hydrocortisone, and 14.5% of patients received methylprednisolone. 

As shown for the secondary line of treatment in Figure 2 Sunburst, 2.10% of burn patients who initially received dexamethasone were switched to triamcinolone. Additionally, 1.20% of those patients were switched to hydrocortisone. In burn patients who initially received triamcinolone, 1.92% were switched to dexamethasone and 0.84% were switched to methylprednisolone. For burn patients who were initially given hydrocortisone, 1.32% were switched to another non-specified medication, and 1.03% were switched to dexamethasone. In burn patients initially given methylprednisolone, 1.27% of those patients were switched to dexamethasone, and 1.19% of patients were changed to a non-specified medication. 

Additionally, 2.02% of all burn patients who were given glucocorticoids were started on a combination of hydrocortisone and triamcinolone. 1.13% of burn patients were started on a combination of methylprednisolone and dexamethasone.

These data suggest that most patients in each group were kept on the same treatment throughout the duration of the study.

### 3.3. Glucocorticoid Treatment Asscociated with Burn Severity in Patients 

#### 3.3.1. 0–19% TBSA Burn

**Figure 3:**
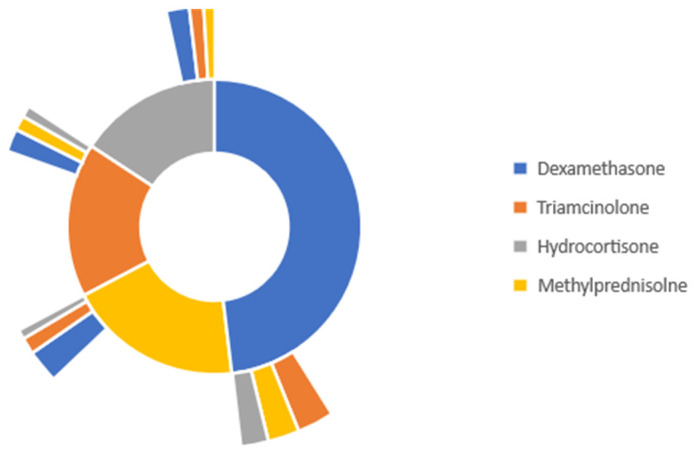
Glucocorticoid treatment pathway in burn patients with 0–19% TBSA burn.

Figure 3 represents the treatment pathway for burn patients with a 0–19% TBSA who were given any of the four specified glucocorticoids. Dexamethasone was the most commonly administered glucocorticoid, and 47.6% of patients were given this medication. Methylprednisolone was the second most common glucocorticoid in 17.2% of burn patients. 15.4% of patients were given triamcinolone, and 14.2% of patients were given hydrocortisone. 

Analysis of secondary lines of treatment reveals that among burn patients with a 0–19% TBSA initially given dexamethasone, 3.13% of those patients were switched to triamcinolone, 2.70% of patients were switched to methylprednisolone, and 2.39% were switched to hydrocortisone. When the same TBSA group was initially started on methylprednisolone, 2.85% of patients were switched to dexamethasone, 1.32% were switched to triamcinolone, and 0.91% were switched to hydrocortisone. For the burn patients started on triamcinolone, 1.91% were switched to dexamethasone, 1.27% were switched to methylprednisolone, and 1.05% were switched to hydrocortisone. Finally, burn patients started with hydrocortisone, 1.93% were switched to dexamethasone, 1.31% were switched to triamcinolone, and 0.86% were switched to methylprednisolone (Figure 3).

#### 3.3.2. 20–39% TBSA Burn

**Figure 4:**
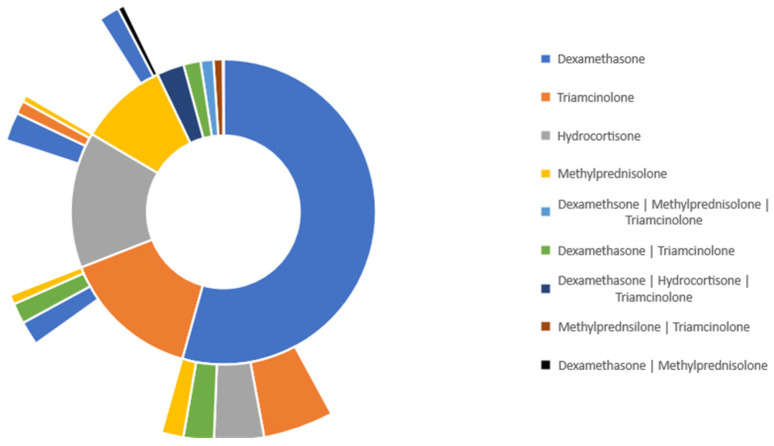
Glucocorticoid treatment pathway among burn patients with 20–39% TBSA.

A similar treatment pathway analysis was conducted for burn patients with a 20–39% TBSA (Figure 4) given either of the four specified glucocorticoids. Dexamethasone was the most commonly administered glucocorticoid, and 52.40% of patients received this specific medication. 13.70% of patients were administered hydrocortisone, and 13.60% of patients were administered triamcinolone. Lastly, methylprednisolone was the least commonly administered glucocorticoid, where only 9.24% of patients initially received this specified glucocorticoid. 

Among burn patients with a 20–30% TBSA who were initially given dexamethasone, 6.15% of patients were switched to triamcinolone, 4.38% of patients were switched to hydrocortisone, 2.69% of patients were switched to a combination of dexamethasone and triamcinolone, and 1.88% of patients were switched to methylprednisolone. For burn patients, they initially started with hydrocortisone, and 2.50% were switched to dexamethasone; 1.18% were switched to triamcinolone; and 0.55% were switched to methylprednisolone. In burn patients initially given triamcinolone, 2.06% were switched to dexamethasone, 1.84% were switched to a combination of dexamethasone and triamcinolone, and 0.81% were switched to methylprednisolone. Lastly, in burn patients who started with methylprednisolone, 1.84% switched to dexamethasone, and 0.59% switched to a combination of methylprednisolone and dexamethasone. 

Additionally, 1.69% of burn patients with a TBSA of 20–39% were started on a combination of dexamethasone, methylprednisolone, and triamcinolone. 2.25% of patients were started on a combination of dexamethasone and triamcinolone. 3.61% of patients were started on a combination of dexamethasone, hydrocortisone, and triamcinolone. Lastly, 1.29% were started on a combination of methylprednisolone and triamcinolone.

#### 3.3.3. 40–100% TBSA Burn

**Figure 5:**
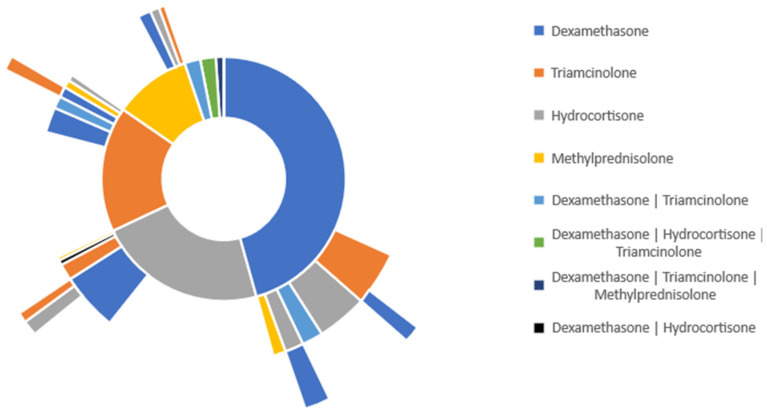
Glucocorticoid treatment pathway in burn patients with 40–100% TBSA.

Figure 5 represents the treatment pathway for patients with a 40–100% TBSA burn who were given either of the four specified glucocorticoids. Dexamethasone was the most administered glucocorticoid in this group, with 43.3% of patients. Hydrocortisone was the second most administered glucocorticoid, with 20.4% of patients. Moreover, 15.1% of patients received triamcinolone, and 10.5% of patients received methylprednisolone. 

Among burn patients who initially received dexamethasone, 6.15% were switched to hydrocortisone, 4.89% were switched to triamcinolone, 2.46% were switched to dexamethasone and triamcinolone, and 1.52% were switched to methylprednisolone. In burn patients who were started on dexamethasone and switched to hydrocortisone, 2.28% of patients were switched back to dexamethasone. Additionally, among the same population of burn patients, 2.86% of patients were started on a combination of dexamethasone and triamcinolone. 2.79% of patients were started on a combination of dexamethasone, hydrocortisone, and triamcinolone. Lastly, 1.40% of patients were started on a combination of dexamethasone, triamcinolone, and methylprednisolone. 

The sunburst illustration of treatment pathways shows the percentage of patients who switched from one treatment to another, probably because of the lowered efficiency of specific treatments when given for a long time or in response to changes in the inflammatory status or treatment indications.

### 3.4. Glucocorticoid Time of Treatment in Overall Burn Patients

**Figure 6:**
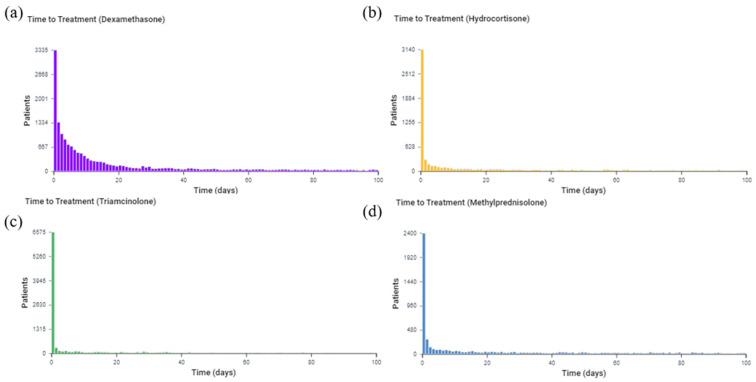
Time of treatment for each glucocorticoid among all burn patients (TBSA unspecified). The *x*-axis represents the observation time from 0 to 100 days after the burn. The *y*-axis is the number of patients receiving treatment at the time. (**a**) Dexamethasone; (**b**) Hydrocortisone; (**c**) Triamcinolone; (**d**) Methylprednisolone.

The analytical tool in TriNetX also allows the determination of the time of treatment pathway for all burn patients who received each of the specified glucocorticoids. The time of treatment analysis in all TBSA burn patients shows that dexamethasone was given for a mean of 56.5 days ± 80.1 days and a median of 13 days (Figure 6). Triamcinolone was given for a mean of 61.3 days ± 84.2 days and a median of 12 days. Hydrocortisone was given for a mean of 65.1 days ± 84.8 days and a median of 19 days. Methylprednisolone was given for a mean of 76.4 days ± 89 days and a median of 36 days.

### 3.5. Does the Time of Treatment for Glucocorticoids Vary Depending on Burn Severity?

#### 3.5.1. 0–19% TBSA Burn

**Figure 7:**
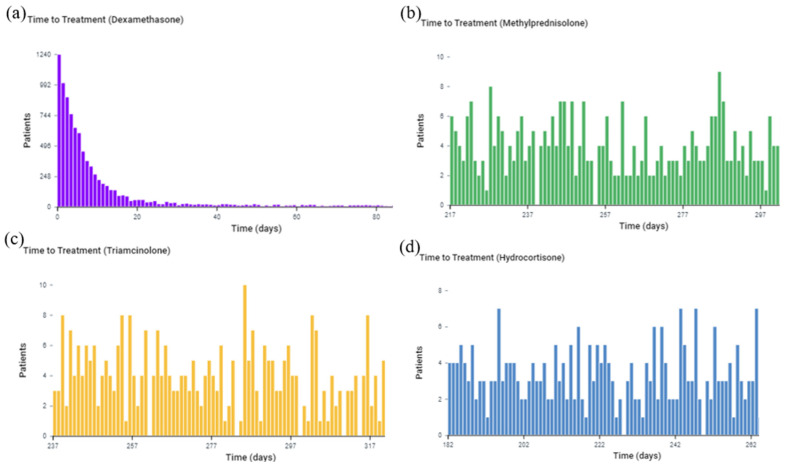
Time of treatment pathway for each glucocorticoid among burn patients with a 0–19% TBSA burn. The *x*-axis is the observation time after the burn. The *y*-axis is the number of patients receiving treatment at the time. (**a**) Dexamethasone; (**b**) Hydrocortisone; (**c**) Triamcinolone; (**d**) Methylprednisolone.

The time of treatment pathway for patients with 0–19% TBSA burn shows that dexamethasone was given for a mean of 490.3 days ± 902.4 days and a median of 28 days. Compared to dexamethasone, triamcinolone was administered to burn patients for a significantly longer period (699.1 days ± 985.7 days, median of 287 days). Similarly, methylprednisolone (mean of 661.2 days ± 954.7 days, median of 267 days) and hydrocortisone (mean of 641.7 days ±934.8 days, median of 232 days) were also given for longer periods compared to dexamethasone. As illustrated in Figure 7, dexamethasone is the drug of choice in the acute phase of burn in this TBSA burn group.

#### 3.5.2. 20–39% TBSA Burn

**Figure 8:**
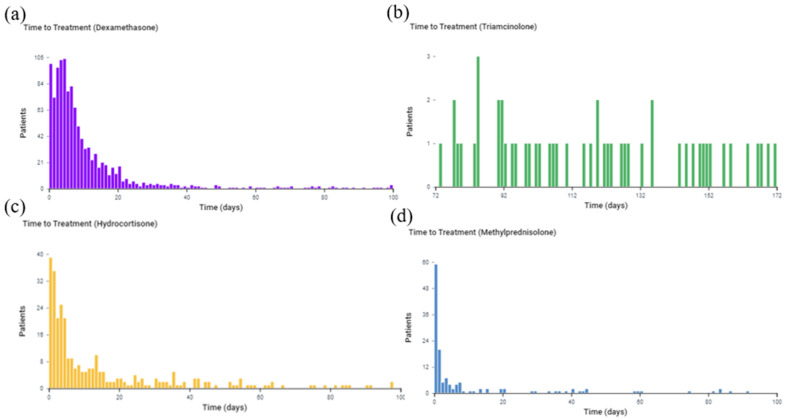
Time of treatment pathway for each glucocorticoid among burn patients with a 20–39% TBSA burn. The *x*-axis represents the observation time from 0 to 100 days after the burn. The *y*-axis is the number of patients receiving treatment at the time. (**a**) Dexamethasone; (**b**) Hydrocortisone; (**c**) Triamcinolone; (**d**) Methylprednisolone.

As seen in Figure 8, dexamethasone (255.6 days ± 757.7 days, median of 8 days) was administered for the shortest period when administered to burn patients with a TBSA of 20–39%. Patients were given hydrocortisone for a mean of 260.1 days ± 734.2 days and a mean of 11 days and given triamcinolone for a mean of 489.6 days ± 971.8 days and a median of 122 days. Methylprednisolone (619.6 days ± 1128 days, median of 42 days) was given for the longest period compared to the other glucocorticoids. Similar to burn patients with a 0–19% TBSA, dexamethasone was the preferred glucocorticoid for the acute phase of burn injury for burn patients with a 20–39% TBSA.

#### 3.5.3. 40–100% TBSA Burn

**Figure 9:**
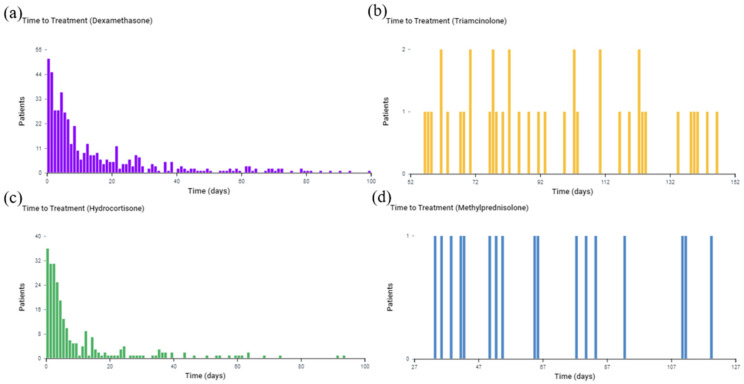
Time of treatment pathway for each glucocorticoid among burn patients with a 40–100% TBSA burn. The *x*-axis represents the observation time from 0 to 100 days after the burn. The *y*-axis is the number of patients receiving treatment at the time. (**a**) Dexamethasone; (**b**) Hydrocortisone; (**c**) Triamcinolone; (**d**) Methylprednisolone.

Hydrocortisone (mean of 213.9 days ± 572.2 days, median of 6 days) was given the shortest duration of time for burn patients with a TBSA of 40–100%, as seen in Figure 9. Patients were given dexamethasone for a mean of 262.5 days ± 694.2 days and a median of 14 days and methylprednisolone for a mean of 292.2 days ± 1207.8 days and a median of 77 days. Triamcinolone (mean of 564.6 days ± 1028 days, median of 102 days) was given for the longest period of time. Unlike the cohorts for burn patients with 0–19% TBSA and 20–39% TBSA, hydrocortisone was the preferred glucocorticoid to be administered acutely for burn patients with a 40–100% TBSA. 

### 3.6. Purity Pathway of Burn Patients Received the Role of Glucocorticoid

**Figure 10:**
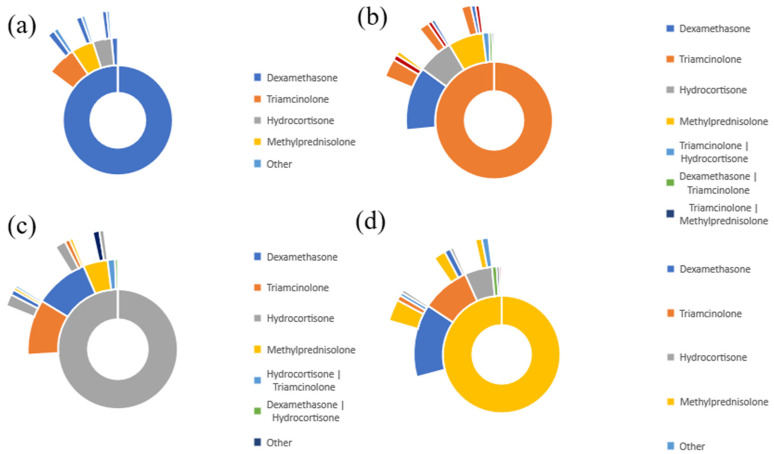
(**a**–**d**) Treatment pathway showing purity for Dexamethasone (**a**), Triamcinolone (**b**), Hydrocortisone (**c**), and Methylprednisolone (**d**).

To determine whether our patients’ cohorts received the specified glucocorticoids exclusively or were assigned to other glucocorticoids at the same time, we analyzed the purity pathway of each glucocorticoid using the treatment pathway analytic tool in TriNetX. Figure 10a–d represents the purity pathway for dexamethasone (a), triamcinolone (b), hydrocortisone (c), and methylprednisolone (d). Each glucocorticoid cohort was determined to have 100% purity. Therefore, no other glucocorticoids were assigned when specific glucocorticoids were analyzed for studying the incidence of hypertrophic scarring. The purity pathway analysis confirmed that each cohort was predominantly assigned a specific treatment as the first line of treatment. 

## 4. Discussion

The purpose of the present work was to use the TriNetX patient database to study the incidence of hypertrophic scarring in burn patients given common glucocorticoids including dexamethasone, methylprednisolone, triamcinolone, and hydrocortisone. The cohort of burn patients used to study the incidence of hypertrophic scarring was categorized based on TBSA burns. Additionally, the TriNetX treatment pathway analytic tool was used to study the distribution of the different glucocorticoids for the treatment of burn patients and if the distribution varied depending on TBSA burn. Lastly, the duration of treatment for each specified glucocorticoid was studied using the time of treatment analytic tool. 

We observed an increased risk of burn patients developing hypertrophic scarring at a TBSA of 0–19% when given hydrocortisone, triamcinolone, and dexamethasone. However, the risk of scarring in the same population was reduced when given methylprednisolone. No significant difference in the risk of scarring was observed in burn patients with a TBSA of 20–39% or 40–100% when given hydrocortisone, triamcinolone, or methylprednisolone. Dexamethasone was the most administered glucocorticoid among all burn patients. Additionally, the time of treatment for glucocorticoids increased as the TBSA burn increased among burn patients. 

Glucocorticoids are commonly given to burn patients to help mitigate the systemic inflammatory response associated with acute burn injury [3]. It has been shown that glucocorticoids given during the acute phase of a burn injury help reduce inflammatory cytokines, leading to a decrease in the incidence of pulmonary infections and stress ulcers [3]. Furthermore, corticosteroids are considered one of the first-line treatments for hypertrophic and keloid scars due to several therapeutic benefits, such as reducing the height and volume of scars as well as reducing associated pain and pruritis [8]. For the treatment of hypertrophic and keloid scars, corticosteroids are most commonly administered intralesionally in the papillary dermis every 2–4 weeks until there is improvement in the scar [9]. Intralesional corticosteroid injections are discontinued when surgery becomes necessary or adverse side effects occur, including hypopigmentation or tissue atrophy [10]. 

There is currently no “gold standard” of clinical care regarding scar management [11]. However, the International Advisory Panel for Scar Management established clinical recommendations based on current clinical evidence to help provide a framework for clinicians. To prevent scar formation in patients who experienced trauma or underwent surgery, it is currently recommended for high-risk patients to receive silicone gel sheeting for 1 month along with intralesional corticosteroid injections for severe cases. High-risk patients are considered patients who experienced hypertrophic or keloid scarring previously or received surgery in areas at risk for scarring [11]. Silicone gel sheeting is considered a first-line treatment for scar prevention in patients who experience widespread burns. However, burn patients often require a combination of treatments, including corticosteroids, silicone gel sheeting, pressure garments, and physical therapy, due to the complexity of burn scars [11]. 

Potential new treatments for hypertrophic scarring after skin damage are under development. In vivo experiments by Zhang et al. demonstrated reduced scar formation using an innovative wound dressing prepared from genipin crosslinked hydrogel networks of carboxymethyl chitosan, poly-γ-glutamic acid, and anti-fibrotic polypeptide [12]. After 28 days of treatment using the novel wound dressing, their study showed collagen type 1 structure and arrangement in the treated groups were close to uninjured skin [12]. The improved organization of collagen fibrils after 28 days of treatment compared to gauze-treated groups is indicative of the anti-scarring capabilities of this innovative wound dressing. Comparatively, our studies showed that certain glucocorticoids, including methylprednisolone, reduced the diagnosis of hypertrophic scarring. A potential mechanism for reduced scar formation by both forms of treatment can be attributed to the inflammatory response being resolved more quickly. However, we found that the degree of effectiveness of corticosteroids to reduce scar daignosis can vary depending on %TBSA. This was not demonstrated in the study by Zhang et al. as their results were derived from in vitro experiments.

Additionally, Zhang et al. showed chitosan-5% hydrogel as another potential new treatment option for hypertrophic scar formation [13]. In vivo, this study demonstrated a higher percentage of wound closure after 14 days in the groups treated with chistosan-5% hydrogel compared to untreated wounds. Wounds treated with chistosan-5% hydrogel with Aloe vera had a smoother and better appearance compared to wounds treated with chistosan-5% hydrogel alone [13]. A possible explanation for the improved wound healing is the impact of the hydrogel on the collagen 1 to collagen 3 ratio. Similarly, our studies also demonstrated improved wound healing, especially for patients given methylprednisolone at a lower %TBSA. However, the chitosan-5% hydrogel may be more beneficial compared to glucocorticoids, as the gel is administered over 14 days while glucocorticoids can be administered for several weeks. Patients may also experience fewer side effects when given a topical gel compared to intralesional injections. 

Another novel method of treatment for hypertrophic scarring is patterned bacterial cellulose wound dressing. Jin et al. demonstrated through in vivo experiments a decrease in scar contraction for groups treated with patterned bacterial cellulose wound dressing compared to control groups after 21 days [14]. The results of our study showed a similar decrease in scar formation for burn patients given certain glucocorticoids based on %TBSA. However, novel wound dressings may be more beneficial for patients due to their rapid decrease in scar formation and minimal side effects compared to glucocorticoids. 

Corticosteroids are frequently administered after hypertrophic scar excision to prevent scar re-formation through a variety of mechanisms [15]. Triamcinolone acetonide is given at a concentration of 10–40 mg/mL every 4–6 weeks until successful scar reduction or if the patient experiences adverse effects. Hydrocortisone is typically administered at a concentration of 25 mg/mL. Methylprednisolone is given at a concentration of 40 mg/mL, and dexamethasone is given at a concentration of 4 mg/mL [16]. Each of these glucocorticoids was given intralesionally [16]. 

Our study showed that methylprednisolone reduced the risk of hypertrophic scarring diagnosis in burn patients. Preclinical studies have shown that steroid injections reduce hypertrophic scarring through various mechanisms, including reducing inflammation, fibroblast growth and proliferation, TGFb1, TGFb2, and platelet-derived growth factor, as well as promoting collagen degeneration [15]. A randomized controlled trial conducted by Manuskiatti et al. showed significant scar flattening for patients given intralesional triamcinolone acetonide at a concentration of 20 mg/mL every 4 weeks for a total of 6 treatments [17]. However, another randomized control trial performed by Berman et al. found that the keloid recurrence rate after post-excisional treatment with intralesional injections of triamcinolone acetonide was 58.5%, compared to a recurrence rate of 51.2% for scars that were not given any treatment. The same study found that there was no significant difference when the concentration of triamcinolone acetonide administered was 10 mg/mL or 20 mg/mL [18]. 

Our study showed that patients with a higher %TBSA burn showed a greater risk of a diagnosis of hypertrophic scarring. This finding is supported by previous studies. In fact, Thompson et al. conducted a prospective study consisting of 300 burn patients and reported a two-fold increase in the risk of hypertrophic scar development in patients with a TBSA burn > 20% compared to patients with a TBSA burn < 20% [19]. 

A limitation of the study is that the TriNetX database does not specify the reason why each glucocorticoid was administered to each burn patient. However, it is important to note that glucocorticoids may impact hypertrophic scarring even when administered for a separate diagnosis. Additionally, the study did not distinguish each glucocorticoid based on the method of administration. Further research should be conducted to determine if a certain method of administration is more effective for the prevention of hypertrophic scarring. Lastly, the TriNetX database does not allow for the analysis of patients who are potentially lost to follow-up, which may lead to a potential bias in the results.

The current treatment, with varied clinical applications, has been investigated. New wound dressings, microneedle transdermals, and nanocarrier transdermals with active components provide novel and feasible delivery routes to optimize the medication effects [20]. In addition to medication, tissue regeneration factors such as mesenchymal stem cells and platelet-rich plasma could speed up wound healing and alleviate scar formation [21]. Drug delivery related to molecular signaling regulation could be the future direction for developing therapeutic approaches [22,23].

## 5. Conclusions

Overall, methylprednisolone was most beneficial in preventing hypertrophic scarring among burn patients independent of TBSA burn, while dexamethasone increased the risk of hypertrophic scarring in burn patients with a TBSA of 20–39% or 40–100%. Dexamethasone was given earlier after a burn injury compared to other glucocorticoids. The findings of our study support the use of certain glucocorticoids as potential therapeutic options to prevent hypertrophic scarring in burn patients. Further studies should be conducted on additional therapeutic options for hypertrophic scarring in burn patients.

## Figures and Tables

**Table 1:** Patient demographics detail the demographic information of all patient groups that received glucocorticoid treatment.

	Hydrocortisone	Methylprednisolone	Dexamethasone	Triamcinolone
Total Number of Patients	5130	3671	15,015	5795
Age of index	31.4 ± 24.6	47.3 ± 19.6	35 ± 22.8	41.2 ± 24.6
Male	2399	1752	8716	2634
Female	2676	1894	6269	3115
White	2689	2495	9477	3449
Black or African American	1400	712	2731	1249
Latino or Hispanic	538	256	1576	567
Asian	133	58	246	170

**Table 2:** Risk ratio of burn patients who received a specified glucocorticoid compared to burn patients who did not receive any glucocorticoid based on TBSA burn. Statistical significance is set at *p* < 0.05.

**Hydrocortisone**			
**TBSA**	**Risk Ratio**	**95% Confidence Interval**	***p*-Value**
0–19%	1.5	1.252–1.833	<0.01
20–39%	1.46	0.965–2.218	0.07
40–100%	1.37	0.697–2.703	0.36
**Triamcinolone**			
**TBSA**	**Risk Ratio**	**95% Confidence Interval**	***p*-Value**
0–19%	1.5	1.283–1.856	<0.01
20–39%	1.36	0.901–2.07	0.14
40–100%	1.31	0.772–2.394	0.37
**Dexamethasone**			
**TBSA**	**Risk Ratio**	**95% Confidence Interval**	***p*-Value**
0–19%	2.93	2.703–3.169	<0.01
20–39%	1.72	1.48–2.008	<0.01
40–100%	1.98	1.508–2.605	<0.01
**Methylprednisolone**			
**TBSA**	**Risk Ratio**	**95% Confidence Interval**	***p*-Value**
0–19%	0.72	0.583–0.89	0.002
20–39%	0.886	0.485–1.62	0.65
40–100%	1.1	0.521–2.584	0.72

## Data Availability

The data utilized in this study was sourced from the TriNetX Federated Data Network, a global platform offering access to deidentified electronic medical records. The authors of this manuscript were granted data access under a specific UTMB-TriNetX agreement. A free academic individual licensing agreement with TriNetX is required to access the underlying data supporting our findings. Interested parties can initiate requests by contacting join@trinetx.com, citing the study and complete list of authors.
